# A potential risk factor of total knee arthroplasty: an infected Baker’s cyst – a case report

**DOI:** 10.1186/s12891-020-3147-2

**Published:** 2020-02-29

**Authors:** Byung-Ill Lee, Jong-Hyeon Seo, Yong-Beom Kim, Gi-Won Seo

**Affiliations:** 1 Departement of Orthopaedic Surgery, Smarton hospital, Bucheon, Korea; 2 Department of Orthopaedic Surgery, Soonchunhyang University Hospital Seoul, Seoul, Korea; 3 Department of Orthopaedic Surgery, Soonchunhyang University Hospital Gumi, 179, 1gongdan-ro, Gumi, Gyeongsangbuk-do 39371 Korea

**Keywords:** Knee joint, Baker’s cyst, Popliteal cyst, Total knee arthroplasty, Case report

## Abstract

**Background:**

In adults, Baker’s cyst development is attributable principally to secondary alterations after degenerative changes. The latter changes often accompany osteoarthritis, and we frequently encounter patients with Baker’s cysts seeking total knee arthroplasty (TKA). Baker’s cysts are not usually subject to extensive preoperative evaluation because the cysts often disappear naturally after surgery, unaccompanied by any adverse symptoms.

**Case presentation:**

A 63-year-old woman presented with moderate pain in the left knee joint that had developed 1 year ago. Posterior knee pain was aggravated on maximum knee flexion. Three months previously, a popliteal mass had become palpable and the patient had undergone needle mass aspiration twice in a local orthopedic hospital, but the mass had recurred. We initially considered TKA for her severe degenerative osteoarthritis. However, we decided to perform only arthroscopic debridement and cyst excision because the patient was experienced severe pain only on maximal knee flexion, and did not want TKA. Pus gushed from the torn cyst during the operation. We diagnosed an infected Baker’s cyst. The patient was treated with a first-generation cephalosporin postoperatively.

**Conclusions:**

A Baker’s cyst that was aspirated and still causes symptoms with altered blood tests needs to be evaluated accurately before TKA.

## Background

Baker’s cyst is the most common benign tumor around knee joints, commonly presenting as an enlarged mass of the bursal sac between the gastrocnemius muscle and the tendon of the semimembranosus muscle in the medial side of the popliteal area. The cyst is associated with degenerative changes of the knee joint, which may cause pain that is not controlled by conservative treatment; total knee arthroplasty (TKA) may be required. In such cases, a Baker’s cyst is not considered to be significant in terms of preoperative evaluation; no complications are encountered in most cases and the cysts may disappear or become smaller after surgery [1].

We encountered a Baker’s cyst infection in a patient with degenerative arthritis associated with moderate knee pain at rest but severe pain at maximum knee flexion. The purpose of this study was to review preoperative evaluation before TKA.

## Case presentation

**Fig. 1:**
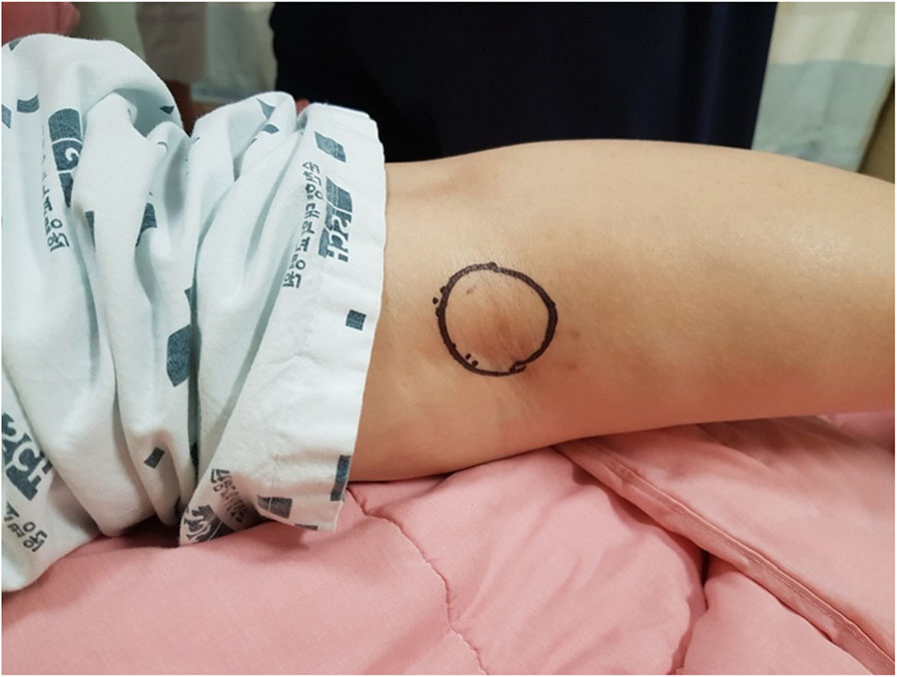
A clinical photograph taken on admission showing mild swelling over the popliteal area

**Fig. 2:**
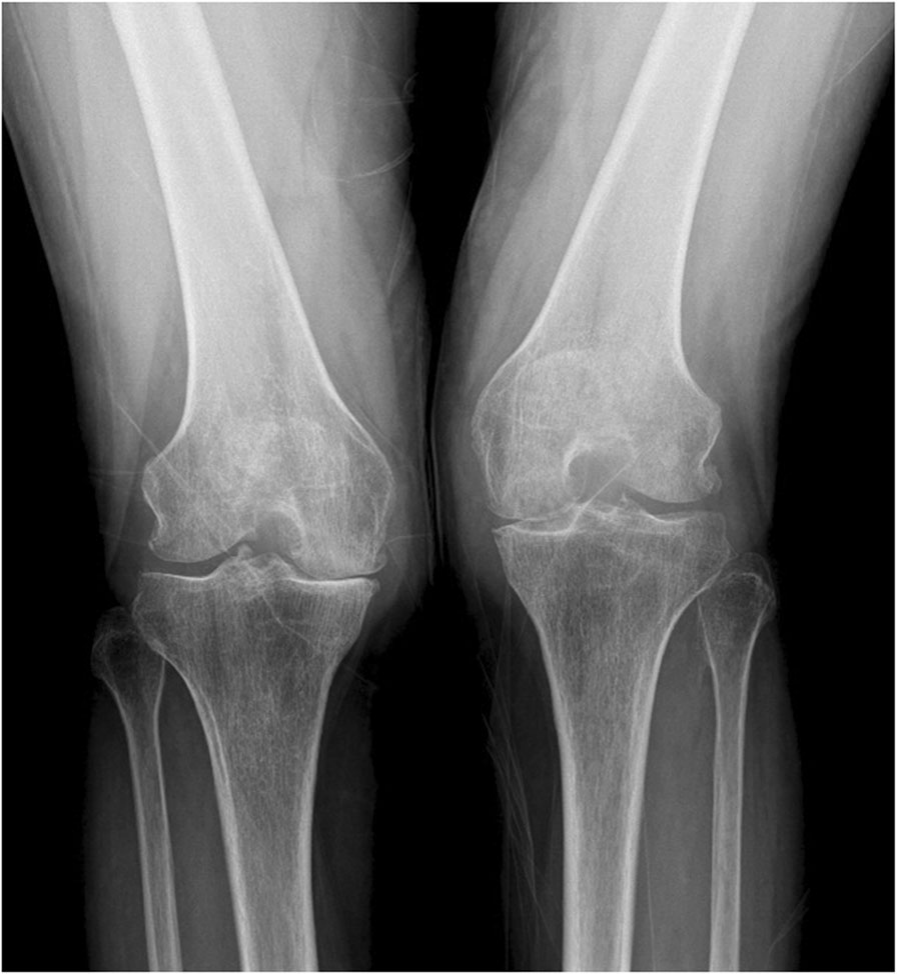
A plain radiograph showing severe osteoarthritis with joint-space narrowing, osteophyte formation, and sclerosis

**Fig. 3:**
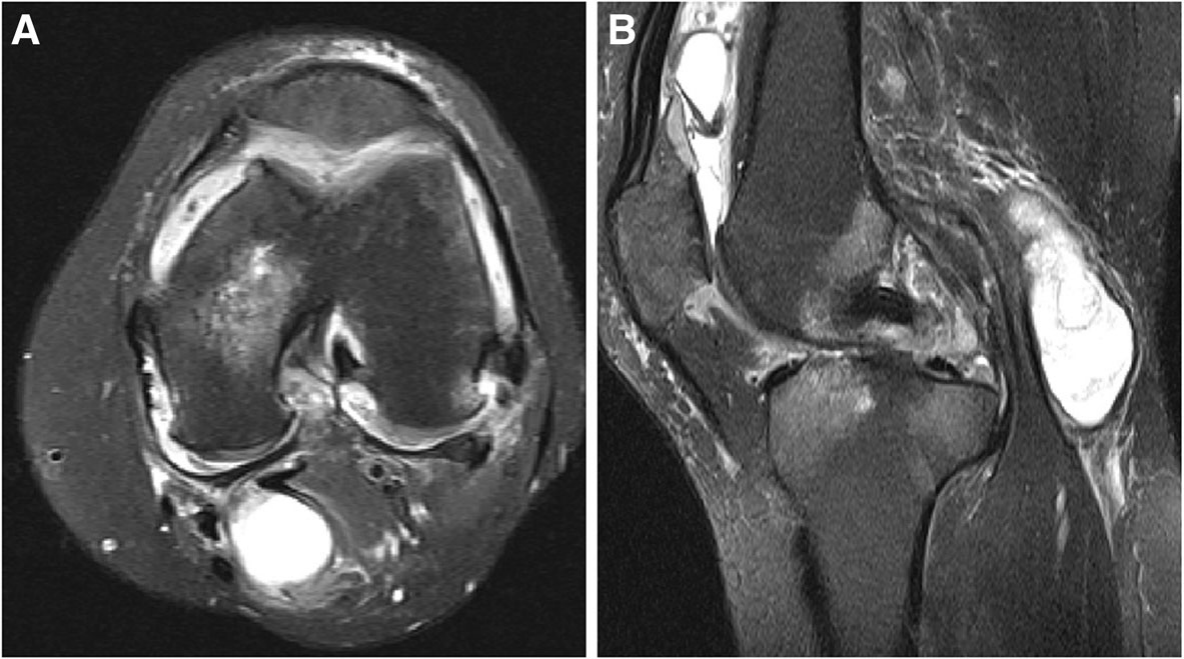
MRI. **a** A T2-weighted, fat suppression axial view showing a high signal-intensity cyst in the popliteal area, communicating with the knee joint. **b** The T2-weighted fat suppression sagittal view shows a cyst within the popliteal area

A 63-year-old woman presented with moderate pain in the left knee joint that had developed 1 year previously. Posterior knee pain became aggravated on maximum knee flexion. Three months previously, a popliteal mass had become palpable, and the patient underwent ultrasound assisted needle mass aspiration (twice) without steroid injection in a local orthopedic hospital. In the outpatient department, the skin was cleaned with 10% povidone-iodine and the site cover with sterile drapes, and the mass was aspirated. No fluid analysis including cell counts or culture was performed and the patient was not prescribed oral or intravenous antibiotics. The patient had no complications, but her symptoms did not change significantly. The mass recurred. Three months of conservative treatment did not produce any improvement. At admission, the range of motion of the left knee was restricted to 10° of flexion contracture and 150° of further flexion. She complained of moderate pain in the left knee at rest but severe pain at maximal flexion. A 2.0 × 2.0-cmmass that was mildly tender was observed in the popliteal area (Fig. 1). No redness or focal warmth was observed around the mass and the patient was not febrile. The following data were recorded on admission: white blood cells 11.2 × 10^3^/μL (neutrophils 72.88%, lymphocytes 19.32%, basophils 0.31%, and eosinophils 1.19%); an erythrocyte sedimentation rate (ESR) of 120 mm/h; and a C-reactive protein (CRP) level of 1.37 mg/dL. *Enterococcus faecium* was isolated from urine, but there were no findings suggesting systemic infection. On plain radiography, osteoarthritis of Kellgren–Lawrence grade 3 (Fig. 2) was confirmed. On T2-weighted magnetic resonance imaging (MRI), a high-signal-intensity cyst was evident on the medial side of the knee, 2.7 × 2.0 × 7.8 cm in dimensions (Fig. 3), connected to both the tendon of the semimembranosus muscle and the medial head of the gastrocnemius muscle. First, TKA was considered based on radiological findings and uncontrolled pain. However, the resting pain was not severe, and the patient did not want TKA; we therefore decided to perform only arthroscopic debridement and Baker’s cyst excision.

**Fig. 4:**
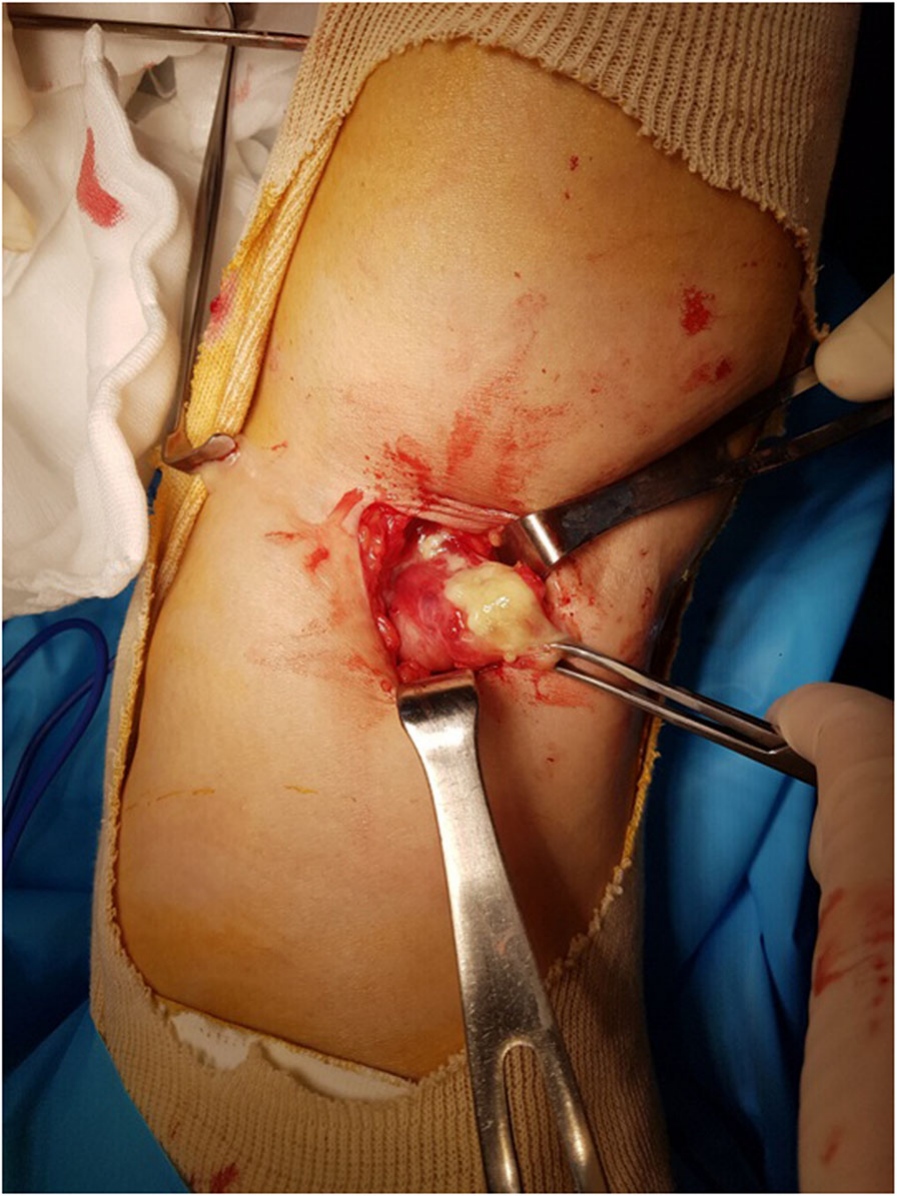
An intraoperative photograph showing yellowish turbid fluid in a Baker’s cyst between the medial head of the gastrocnemius muscle and the tendon of the semimembranosus muscle

Arthroscopy was performed through anterolateral and anteromedial portals with the patient in the supine position. A 1.5 × 1.5-cm osteochondral defect on the medial femoral condyle, and knee inflammation, were evident. We thus performed arthroscopic microfracture and synovectomy. Open excision was chosen because we were unable to find the orifice of the Baker’s cyst at arthroscopy. The patient was then placed in the prone position and the Baker’s cyst excised. A curvilinear skin incision was created in the popliteal area and dissection was performed between the semimembranosus muscle and the medial head of the gastrocnemius muscle. Granulation tissue scattered around the cystic mass was removed. During cyst excision, the cyst tore and yellowish pus-like material flowed out (Fig. 4). Intraoperative culture tests were negative. Histological examination revealed acute and chronic nonspecific inflammation, granulation tissue formation and fibrin deposition. The patient was treated postoperatively with a first-generation cephalosporin (cefazolin) because the organism most commonly causing Baker’s cyst infection is *Staphylococcus aureus* [2]. The symptoms had improved 6 weeks after surgery.

## Discussion and conclusion

Baker’s cyst was first reported by Adams in 1840, in detail by Baker in 1877, and in even more detail in various later articles. Infection of a Baker’s cyst is very rare (21 reported cases) [3]. Most such infections developed in immunosuppressed patients, such as those with myelodysplastic syndrome, psoriasis, rheumatoid arthritis, or Epstein-Barr virus infection [2, 4].

On the first postoperative day, the CRP was 9.62 mg/dL and it decreased to 2.67 mg/dL by 2 weeks postoperatively. The ESR remained at 120 mm/h continuously. Two months postoperatively, the patient had no knee symptoms, but the CRP had increased to 4.07 mg/dL. During evaluation of the cause, rheumatoid arthritis was diagnosed based on a 2010 American College of Rheumatology/European League Against Rheumatism classification criteria total score of 7. However, even with a diagnosis of rheumatoid arthritis and planned TKA, we would not have considered evaluating a Baker’s cyst infection.

The clinical manifestations of a Baker’s cyst are ischemic pain, and claudication caused by compression of the popliteal artery if the cyst is large. Also, knee joint movement is limited. To date, no symptoms distinguishing a typical Baker’s cyst from an infected cyst have been described. Rupture of an infected Baker’s cyst can trigger signs of infection, and symptoms similar to those of deep vein thrombosis may develop because of pain in the popliteal area [3]. To confirm infection of a Baker’s cyst, fine-needle aspiration is required. The aspiration fluid is usually synovial fluid-like in appearance, but the fluid from an infected Baker’s cyst is characteristically pyogenic in appearance [5]. However, invasive examination of the surgical site is generally avoided by clinicians planning TKA.

Periprosthetic joint infection occurs in 0.8–1.9% of patients after TKA [6]. Periprosthetic joint infection requires a long hospital stay incurring major costs, upsetting both the clinician and patient. Thus, before TKA, it is important to conduct a careful preoperative assessment to prevent postoperative infection. Kong et al. [7] reported that age, obesity, operation time, drain usage, diabetes mellitus, urinary tract infection, and rheumatoid arthritis were risk factors for periprosthetic joint infection. Ratto et al. [6] reported that careful management of glucose level, general nutrition and body weight, as well as smoking cessation, reduced infection rates. However, Baker’s cysts may disappear naturally after surgery and, even if they persist, no clinical manifestations are evident. Thus, clinicians usually do not treat a cyst or evaluate it in detail preoperatively.

In this case, had we performed the TKA as initially planned, periprosthetic joint infection was inevitable. Of course, if the CRP level is high, the preoperative assessment is more rigorous, but it may remain difficult to identify an infected Baker’s cyst. If rheumatoid arthritis had been diagnosed preoperatively, we would have attributed the elevated CRP to this, and would have proceeded with TKA associated with a very high risk of periprosthetic joint infection.

Although generalization is impossible given that we describe only one case, patient evaluation before TKA requires a detailed examination of a previously aspirated Baker’s cyst that remains symptomatic, especially in the presence of altered blood tests, given the potential severity of complications if the cyst is infected.

## Data Availability

Not applicable.

## Abbreviations

CRP: C-reactive protein
ESR: Erythrocyte sedimentation rate
TKA: Total knee arthroplasty
